# Operational Stability, Regenerability, and Thermodynamics Studies on Biogenic Silica/Magnetite/Graphene Oxide Nanocomposite-Activated *Candida rugosa* Lipase

**DOI:** 10.3390/polym13213854

**Published:** 2021-11-08

**Authors:** Adikwu Gowon Jacob, Roswanira Abdul Wahab, Mailin Misson

**Affiliations:** 1 Department of Chemistry, Faculty of Science, Universiti Teknologi Malaysia (UTM), Johor Bahru 81310, Johor, Malaysia; gowon.adikwu.jacob@graduate.utm.my; 2 Department of Applied Chemistry, Federal University Dutsin-Ma (FUDMA), Dutsin-Ma P.M.B. 5001, Katsina State, Nigeria; 3 Enzyme Technology and Green Synthesis Group, Faculty of Science, Universiti Teknologi Malaysia (UTM), Johor Bahru 81310, Johor, Malaysia; 4 Advanced Membrane Technology Research Centre (AMTEC), Universiti Teknologi Malaysia (UTM), Johor Bahru 81310, Johor, Malaysia; 5 Biotechnology Research Institute, Universiti Malaysia Sabah, Jalan UMS, Kota Kinabalu 88400, Sabah, Malaysia

**Keywords:** graphene oxide, nanocomposite, operational stability, thermodynamics, esterification synthesis, ethyl valerate, biocatalyst characterization

## Abstract

Inorganic biopolymer-based nanocomposites are useful for stabilizing lipases for enhanced catalytic performance and easy separation. Herein, we report the operational stability, regenerability, and thermodynamics studies of the ternary biogenic silica/magnetite/graphene oxide nanocomposite (SiO_2_/Fe_3_O_4_/GO) as a support for *Candida rugosa* lipase (CRL). The X-ray photoelectron spectroscopy (XPS), X-ray diffraction (XRD), field-electron scanning electron microscopy (FESEM), vibrating sample magnetometry (VSM), and nitrogen adsorption/desorption data on the support and biocatalyst corroborated their successful fabrication. XPS revealed the Fe_3_O_4_ adopted Fe^2+^ and Fe^3+^ oxidation states, while XRD data of GO yielded a peak at 2θ = 11.67°, with the SiO_2_/Fe_3_O_4_/GO revealing a high surface area (≈261 m^2^/g). The fourier transform infrared (FTIR) spectra affirmed the successful fabricated supports and catalyst. The half-life and thermodynamic parameters of the superparamagnetic immobilized CRL (CRL/SiO_2_/Fe_3_O_4_/GO) improved over the free CRL. The microwave-regenerated CRL/SiO_2_/Fe_3_O_4_/GO (≈82%) exhibited higher catalytic activity than ultrasonic-regenerated (≈71%) ones. Lower activation ($E_{a})$ and higher deactivation energies ($E_{d})$ were also noted for the CRL/SiO_2_/Fe_3_O_4_/GO (13.87 kJ/mol, 32.32 kJ/mol) than free CRL (15.26 kJ/mol, 27.60 kJ/mol). A peak at 4.28 min in the gas chromatograph-flame ionization detection (GC-FID) chromatogram of the purified ethyl valerate supported the unique six types of 14 hydrogen atoms of the ester (CAS: 539-82-2) in the proton nuclear magnetic resonance (^1^H-NMR) data. The results collectively demonstrated the suitability of SiO_2_/Fe_3_O_4_/GO in stabilizing CRL for improved operational stability and thermodynamics and permitted biocatalyst regenerability.

## 1. Introduction

Unlike immobilized lipases, individual carrier/enzyme catalysts are highly vulnerable to agglomeration, adversely influencing their activity and stability. Therefore, the graphene-based magnetic silica nanocomposite combination shows good prospects as a greener and sustainable biopolymer resource. This is given the nanocomposite’s ability to stabilize lipases for higher catalytic activity and operational stability, as well as easier separation for recycling [1,2,3]. Moreover, the benefits of inorganic biopolymer-based support come from its network’s large specific surface area. The network also protects the attached lipase against physical, chemical, and microbial attacks. The abundant silanol (Si-OH) and siloxane (Si-O-Si) groups at the supports’ surface facilitate the effective binding of enzymes to the carriers [4,5,6]. Using graphene oxide (GO) and magnetite (Fe_3_O_4_) as support components can reduce agglomeration and improve the mechanical and operational stability of immobilized CRL [1,7,8]. The oxygenated functional groups in GO form additional multipoint covalent bonds within the nanocomposite, while the magnetite’s paramagnetism facilitates biocatalysts recovery [1]. Such characteristics in enzyme supports are vital in overcoming the above-said issues.

In the beginning, binary nanocomposites were the popular support choice for preparing immobilized lipases [4,5]. A study previously employed the *Rhizomucor miehei* lipase supported on a chitosan/graphene oxide nanocomposite capable of catalyzing the synthesis of 49.46% geranyl propionate [9]. Elias et al. [10] successfully yielded a higher quantity of butyl butyrate at 76.30% using CRL supported on a chitosan/nanocellulose nanocomposite. By comparison, Onoja et al. [8] recorded 94.0% of butyl butyrate using CRL conjugated to a silica–magnetite nanocomposite. The ternary composites exhibited good operational stability and enzyme activity over the binary composite-immobilized enzymes.

The current immobilization trend considers ternary nanocomposites as better supports for lipase stabilization and biocatalyst recycling by incorporating magnetite nanoparticles [11]. For instance, the ternary chitosan/chitin/Fe_3_O_4_ covalently bound CRL produced 96% pentyl valerate and retained ≈90% initial activity after 40 days of storage [12]. Another similar study using lipase conjugated to alginate/nanocellulose/montmorillonite composite produced 92.89% ethyl levulinate in just 6 h. The immobilized lipase also exhibited better thermostability than its unbound counterparts [13]. In another study, CRL bound on the Fe_3_O_4_/SiO_2_/PAMAM nanocomposite was reusable for seven successive reaction cycles, retaining 68% residual activity [14]. Despite these advancements, the technology to fabricate novel ternary nanocomposites for lipase immobilization is still in its infancy. Much is needed to improve the immobilization procedure. Further studies are necessary to gauge the efficacy extent of different nanocomposites to improve the stability and activity of immobilized enzymes. Pertinently, the CRL (hydrolases EC 3.1.1.3) immobilized onto the nanocomposite developed in this study is a versatile enzyme capable of synthesizing high yields of several types of ester [1,2,3,4,5,6]. One major problem is that free CRL is susceptible to deactivation under elevated temperature and pH. The lipase also exhibits low activity in non-aqueous solvents, shows low operational stability, and biocatalyst recovery is difficult [12]. Immobilizing the free CRL on hydrophobic nanocomposites might circumvent these drawbacks [8,9,10,11,12,13].

Ethyl valerate (EV) also has applications in the pharmaceutical, food, detergents, and cosmetics industries and shows compatibility as biofuel or additive for diesel and gasoline engines. However, the ester is currently produced primarily via synthetic chemical routes or by extraction [1], invoking concerns on safety and sustainability issues. Such routes are undesirable because of the high-energy input, liberation of large quantities of greenhouse gases and unwanted by-products, low extraction yields, and high labor costs [1]. A biotechnological production route to manufacture EV could circumvent these problems [1,8,15], using immobilized-lipase catalyzed esterification reactions [16,17,18,19,20,21,22]. Thus, the biocatalyst’s strengths and limitations should be identified, as the latter affects production costs. Herein, this study examines the influence of biogenic SiO_2_/Fe_3_O_4_/GO nanocomposites on the operational stability and thermodynamics of CRL/SiO_2_/Fe_3_O_4_/GO compared to free CRL. The regenerability of the spent catalyst was also evaluated, including thermal- and short-term storage stability tests. The half-life, activation energy, rate constant, enthalpy, entropy, and free energy of denaturation of both lipases were assessed under the established optimized conditions. Moreover, the extent to which the SiO_2_/Fe_3_O_4_/GO nanocomposite affects the immobilized CRL stability and catalytic property for EV production remains unreported. The obtained information could offer new insights into the SiO_2_/Fe_3_O_4_/GO nanocomposite’s feasibility as support for other types of enzymes.

## 2. Materials and Methods

### 2.1. Materials and Chemicals

Raw oil palm leaves were obtained from an oil palm plantation at Universiti Teknologi Malaysia. Hydrochloric acid (HCl), sodium hydroxide (NaOH), dipotassium hydrogen phosphate (K_2_HPO_4_), potassium dihydrogen phosphate (KH_2_PO_4_), and ethyl acetate were purchased from QReC Chemicals (Auckland, New Zealand), whereas graphite and glutaraldehyde (25 wt.%) were respectively acquired from Fluka Chemicals (Glossop, UK) and ACROS Organics (Pittsburgh, PA, USA). Ammonia, toluene, iron (III) chloride hexahydrate (FeCl_3_·6H_2_O), iron (II) chloride tetrahydrate (FeCl_2_·4H_2_O), sodium nitrate (NaNO_3_), sulfuric acid (H_2_SO_4_), glacial acetic acid (99%), potassium permanganate (KMnO_4_), hydrogen peroxide, *n*-hexane, *n*-heptane, and ethanol were bought from Merck (Darmstadt, Germany). Bradford reagent and methanol were provided by Vchem Chemicals (Gujarat, India). *Candida rugosa* lipase Type VII (≥700 U/mg), valeric acid, 3-aminopropyltriethoxysilane (99%), 3 Å molecular sieves, and cetyltrimethylammonium bromide (CTAB) were bought from Sigma-Aldrich (Steinheim am Albuch, Germany). All chemical reagents used were of analytical grade.

### 2.2. Preparation of Support Systems

#### 2.2.1. Preparation of Biogenic Silica, Graphene Oxide, and Magnetic Graphene Oxide

The components of the nanocomposite support comprising biogenic silica, graphene oxide, and magnetic graphene oxide were prepared according to our recently published method [1]. The graphene oxide (GO) was prepared from graphite, as previously described by Ranjbari et al. [23], and the Fe_3_O_4_/GO hybrid composite was synthesized via a modified co-precipitation. In this study, 5 g/mL of GO, FeCl_2_·4H_2_O (0.006 mol), FeCl_3_·6H_2_O (0.013 mol) (molar ratio of 1:2), and acetic acid solution (100 mL) were transferred into a three-necked round-bottom flask (250 mL) [24]. The mixture was stirred for 1 h at room temperature before raising the temperature to 90 °C. Next, concentrated ammonia (20 mL, 35% *w/v*) was added dropwise into the mixture with rapid stirring at 500 rpm for 25 min to precipitate the Fe_3_O_4_/GO. The resultant nanocomposites were rinsed successively in copious amounts of deionized water and methanol before drying at 60 °C for 10 h. The subsequent co-precipitation reaction transpired under the N_2_ atmosphere to prevent critical oxidation.

#### 2.2.2. Preparation and Modification of the SiO_2_/Fe_3_O_4_/GO Nanocomposite

**Scheme 1:**
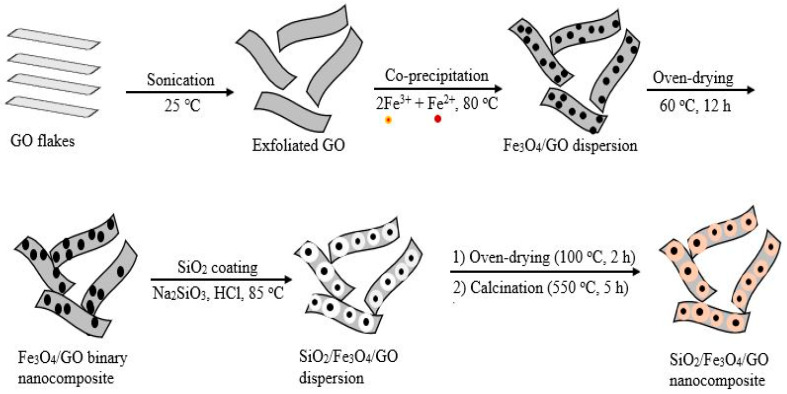
Schematic illustration for the preparation of SiO_2_/Fe_3_O_4_/GO ternary nanocomposite.

The core-shell of the ternary SiO_2_/Fe_3_O_4_/GO nanocomposite was prepared as previously reported [6], with slight modifications when growing the silica (SiO_2_) layers over the Fe_3_O_4_/GO surface to fabricate the SiO_2_/Fe_3_O_4_/GO [1]. In this study, 1.0 g of Fe_3_O_4_/GO was suspended in 5 mL of deionized water containing HCl (1 mL, 6.0 M), and the mixture was sonicated for 15 min. Next, the sodium silicate (Na_2_SiO_3_) solution (25 mL, 0.271 mol) was acidified using HCl (1 mL, 6.0 M) to pH 12 before adding to the Fe_3_O_4_/GO suspension. The suspension was sonicated for 10 min at 30 °C, and CTAB solution (1.0 g per 40 mL of deionized water) was added with stirring (300 rpm) before raising the temperature to 85 °C. Ethyl acetate (3.5 mL) was added to the suspension, stirred (500 rpm) for a further 15 min, and then cooled to room temperature. The suspension’s pH was reduced to 6.5 using HCl (3.0 M) and was stirred (300 rpm) further for 12 h and left to age overnight at ambient temperature. The as-prepared SiO_2_/Fe_3_O_4_/GO nanocomposite was magnetically separated and thoroughly rinsed with deionized water until the washings reached pH 7.0. Finally, the suspension was oven-dried (100 °C, 2 h) and calcined (550 °C, 5 h). Next, surface –NH_2_ groups were introduced to the SiO_2_/Fe_3_O_4_/GO nanocomposite using APTES. It was followed by an activation process using glutaraldehyde as the crosslinking agent to afford the GL-A-SiO_2_/Fe_3_O_4_/GO nano support [25]. As a result of the instability of the aldehyde group, CRL immobilization was immediately done by stirring the GL-A-SiO_2_/Fe_3_O_4_/GO nano support in 3 mg/mL of the lipase solution [1,6]. Finally, the Bradford assay quantified the resultant CRL/SiO_2_/Fe_3_O_4_/GO biocatalyst [26]. Scheme 1 illustrates the preparation of the SiO_2_/Fe_3_O_4_/GO ternary nanocomposite.

### 2.3. Esterification Synthesis of EV Catalyzed by CRL/SiO_2_/Fe_3_O_4_/GO

An esterification mixture comprising valeric acid and ethanol (300 μL, molar ratio 1:2), and 3 mg/mL of CRL/SiO_2_/Fe_3_O_4_/GO or free CRL in n-heptane (3.7 mL) were mixed in a glass vial (20 mL) before stirring (200 rpm) in an oil bath at 40 °C. After the second hour, an appropriate amount of molecular sieves (4 Å in diameter, 8–12 mesh) was added to the reaction mixture. Regular sampling of the reaction mixture was carried out according to our recently reported study [27]. The enzymatically produced EV was evaluated as the percentage of valeric acid converted over the total valeric acid (Equation (1)):

(1) $$\mathrm{Ester}\;\mathrm{conversion}\;\left(\%\right)=\left(\frac{V_{o}-V_{t}}{V_{o}}\right)\times 100$$

where $V_{o}$ and $V_{t}$ are the initial volumes (mL) of NaOH at time (t = 0) and time (t = 3), respectively. The experiments were triplicated to minimize error, and each result is presented as mean ± standard deviation.

### 2.4. Characterization of Support Matrix and Biocatalyst

#### 2.4.1. Chemical Composition, Oxidation States, and Microcrystalline Structure Analysis

X-ray photoelectron spectroscopy (XPS) study of Fe_3_O_4_ nanoparticles, SiO_2_/Fe_3_O_4_/GO, and CRL/SiO_2_/Fe_3_O_4_/GO for the elemental composition and oxidation states of iron were done on a high-resolution Auger electron spectrometer-AXIS ULTRA (Kratos Analytical, Shimadzu, Manchester, UK), equipped with monochromatic Al kα. The CasaXPS software was used to process the collected data. X-ray diffractograms (XRD) of crystalline points for graphite and synthesized GO structures were obtained on a D/max 2200 (Rigaku, Tokyo, Japan) diffractometer. The instrument was operated at a Cu Kα radiation source of λ = 1.506 Å, under 40 kV and 30 mA, using an angular range of 2θ = 10° to 70°.

#### 2.4.2. Textural Properties and Pore Structure Analysis

The GL-A-SiO_2_/Fe_3_O_4_/GO nano support texture was analyzed on a Micromeritics (3Flex 3.01; Norcross, GA, USA) nitrogen adsorption/desorption at 77 K. Before the test, approximately 100 mg of the sample was degassed at 80 °C for 2 h. The surface area was evaluated using a Brunauer-Emmett-Teller (BET) method at relative pressures (*P/P_o_*) between 0.05 and 0.30. The total pore volume was estimated from the quantity of nitrogen adsorbed at *P/P_o_* near 1. Meanwhile, the Barret-Joyner-Halenda (BJH) method estimates the pore size using the desorption branches of the isotherms, and the average pore diameter (D_P_) was approximated using Equation (2) [28].

(2) $${\;D}_{p}\;\left(\mathrm{nm}\right)=4\times 10^{3}\;\frac{\mathrm{Pore}\;\mathrm{volume}\;\left({\mathrm{cm}}^{3}/g\right)}{\mathrm{Surface}\;\mathrm{area}\;\left(m^{2}/g\right)}$$

#### 2.4.3. Surface Morphological and Microstructural Analysis

The field-electron scanning electron microscope (FESEM, JSM-6700F, JEOL, Tokyo, Japan) was used to examine the morphological properties of the ternary SiO_2_/Fe_3_O_4_/GO composite and the immobilized catalyst (CRL/SiO_2_/Fe_3_O_4_/GO). The samples were subjected to an accelerating voltage and current at 5 kV and 10 μA, respectively. Each silicon wafer surface-mounted sample was sputter-coated with a thin film of gold to prevent charging under the electron beam. Meanwhile, a scanning electron microscope (SEM) (JSM-IT300, JEOL, Tokyo, Japan) operating at 30 kV accelerating voltage was used to analyze the surface morphologies and structures of the reused and regenerated CRL/SiO_2_/Fe_3_O_4_/GO.

#### 2.4.4. Magnetic Behavior Analysis

The Fe_3_O_4_, Fe_3_O_4_/GO, SiO_2_/Fe_3_O_4_/GO, and CRL/SiO_2_/Fe_3_O_4_/GO samples were subjected to saturation magnetization on a vibrating sample magnetometer (Lakeshore VSM 7404, Cryotronics, Westerville, OH, USA). A powder sample (0.3 mg) was placed on a sample holder (nickel rod) and rotated in a vibration exciter under a magnetic field from zero to 3.5 T, at a frequency of 85 Hz for 60 min. Sample readings were taken for temperatures between −196 and 900 °C and magnetic moments from 10.2 to 300 emu. The relative accuracy was 0.1 mT at ±1% resolution.

#### 2.4.5. Chemical Composition and Functional Group Analysis

Fourier transform infrared (FTIR) spectroscopy analysis was performed using a Perkin-Elmer spectrophotometer (Frontier 100; Waltham, MA, USA) in one-bounce attenuated total reflectance (ATR) mode. The spectra were acquired in transmission mode for wavelengths between 400 and 4000 cm^−1^ at 16 scans and 4 cm^−1^ resolution.

### 2.5. Effect of Reaction Time on Esterification of Ethanol and Valeric Acid

The effect of reaction time for both free CRL and CRL/SiO_2_/Fe_3_O_4_/GO-catalyzed reactions was assessed between 1 and 6 h. Each reaction comprised each biocatalyst (3 mg/mL) dissolved *n*-heptane, valeric acid, and ethanol molar ratio (1:2) and was stirred (200 rpm) at 40 °C. The enzymatically produced EV was quantified from triplicated reactions, using Equation (1) described in Section 2.3.

### 2.6. Operational Stability Studies

#### 2.6.1. Thermal Stability

The thermal stability of free CRL compared to CRL/SiO_2_/Fe_3_O_4_/GO used a previously described modified method [13]. Each biocatalyst was transferred into a vial containing *n*-heptane and pre-incubated in a paraffin oil bath for 1 h at various temperatures (20−70 °C). Then, the substrates (valeric acid/ethanol, ratio 1:2) were added to the reaction systems and magnetically stirred (200 rpm) for 3 h before titration with NaOH (0.2 M) to estimate the produced EV (Equation (1)).

#### 2.6.2. Half-Life (T_50_)

The free CRL (3 mg/mL) and CRL/SiO_2_/Fe_3_O_4_/GO (3 mg/mL) were incubated in *n*-heptane at 50 °C for 120 h to establish their T_50_. The reaction mixtures were sampled according to a reported method by Onoja et al. [8]. The T_50_ of each lipase was considered at 50 °C, at a temperature higher than the optimum (40 °C).

#### 2.6.3. Storage Stability

The storage stability of CRL/SiO_2_/Fe_3_O_4_/GO and the free CRL was studied as described in the literature [12], with slight modifications. Both biocatalysts were stored at room temperature and their corresponding optimal reaction temperatures. Then, the samples were assayed for the esterification synthesis of EV at regular five-day intervals for 30 days. The esterification activity is presented as percentage relative conversions of valeric acid to EV, taking the first-day ester quantity as 100%.

#### 2.6.4. Regeneration Study

The regenerability of CRL/SiO_2_/Fe_3_O_4/_GO was assessed by subjecting the spent biocatalyst to (i) ultrasonic and (ii) microwave treatments. Briefly, the ultrasonication regenerated CRL/SiO_2_/Fe_3_O_4/_GO was immersed in *n*-heptane at room temperature and ultrasonicated continuously for 1–5 min durations. For the microwave treatment, the spent CRL/SiO_2_/Fe_3_O_4/_GO was dispersed in sodium phosphate buffer (100 mM, pH 7.0) and microwaved for 5 min at 200 W at 50 °C. After cooling, the biocatalyst was magnetically recovered, rinsed with *n*-heptane, and dried in a desiccator for 6 h. The regenerated biocatalyst was resuspended in a fresh medium of valeric acid/ethanol (ratio1:2) and assayed under optimal conditions, as described in Section 2.6. The mixture was titrated to estimate the quantity of the produced EV (Equation (1)).

### 2.7. Thermodynamic Study-Effect of Temperature on Free CRL and CRL/SiO_2_/Fe_3_O_4_/GO

The method by Dhiman et al. [29] was adopted with minor modification to compare the thermal denaturation and thermodynamic parameters, such as activation and activation energy of free CRL and CRL/SiO_2_/Fe_3_O_4_,/GO. The effect of temperature on both lipases was tested between 20 and 50 °C, where their lipase activities were monitored for 3 h. The first-order rate constants ($k_{a}$) were calculated using Equations (3) and (4):

(3) $$ln\frac{{\left[A\right]}_{0}}{{\left[A\right]}_{t}}=kt\;\;$$

(4) $$ln{\left[A\right]}_{t}=-kt+\ln{\left[A\right]}_{0}$$

where [*A*]_0_ and [*A*]*_t_* represent the concentrations of valeric acid at times (0 h) and specific time (*t*), respectively, *t* = reaction time, and *k* = kinetic constant (temperature dependent). The activation energy ($E_{a}$) of the CRL/SiO_2_/Fe_3_O_4_/GO-catalyzed reaction was estimated from the $k_{a}\;$ values. $E_{a}$ is the minimum energy of activation, whereas $E_{a}$ refers to the esterification synthesis of EV by the free CRL and CRL/SiO_2_/Fe_3_O_4_/GO, determined from Arrhenius plots, $ln\;k_{a\;}$ against T × $10^{–4}$ (Equations (5) and (6)):

(5) $$k_{0}=A_{0}\;\exp\;\left(-\frac{E_{a}}{RT}\right)$$

(6) $$E_{a}=-\;\mathrm{slope}\;\times \;R\;$$

where $k_{0}$ = initial specific rate constant, $A_{0}$ = pre-exponential factor or interaction frequency of the molecules, and exp ($E_{a}$−/*RT*) = minimum energy needed for molecules to interact. R = molar gas constant (8.314 J mol^−1^ K^−1^), and *T* is the absolute temperature in Kelvin (K).

Enzymatic activity decreases with increasing temperatures above their optimums, causing enzyme unfolding followed by deactivation. The study examined the enzyme deactivation, *E_d_*, expressed at a rate that follows an irreversible first-order kinetic and is directly proportional to the catalytic activity (Equation (7)):

(7) $$\frac{dA}{dt}=-kd\times A$$

where *A* = the enzyme activity, *t* = incubation time, and $k_{d}$ = first-order deactivation constant.

The $k_{d}\;$ values for CRL/SiO_2_/Fe_3_O_4_/GO were assessed at a temperature (*T*) between 45 and 70 °C from slopes of the regressions by plotting the experimental values of ln $A_{res}$/$A_{0}$ ($A_{res}$: residual enzyme activity, $A_{0}$: initial enzyme activity) against time (min). The deactivation energy ($E_{d}$) for CRL/SiO_2_/Fe_3_O_4_/GO was estimated using Equation (7) by replacing $k_{0}$ with $k_{d}$ for each temperature. The slope was determined by plotting ln $k_{d}$ versus temperature (K). The half-life ($t_{1/2}$) of CRL/SiO_2_/Fe_3_O_4_/GO refers to the duration when the enzymatic activity reduces to half of its initial, as estimated using Equation (8):

(8) $$t_{1/2}\;=\frac{\ln2}{k_{d}}$$

The decimal reduction time (D-value) refers to the time required for a 10-fold decrease in initial enzyme activity at a given temperature (Equation (9)), while the stabilization factor (*SF*) is estimated using Equation (10):

(9) $$D-\mathrm{values}\;=\frac{\ln10}{k_{d}}$$

(10) $$SF=\frac{t_{1/2}^{iCRL}}{t_{1/2}^{fCRL}}\;\;\;$$

where *iCRL* denotes the CRL/SiO_2_/Fe_3_O_4_/GO and fCRL represents the free CRL.

For thermal deactivation, Equations (11)–(13) compute the thermodynamic parameters concerning changes in standard enthalpy ($\Delta H_{d}^{{}^{\circ}}$), standard entropy ($\Delta S_{d}^{{}^{\circ}}$), and Gibb’s free energy ($\Delta G_{d}^{{}^{\circ}}$):

(11) $$\Delta H_{d}^{{}^{\circ}}=E_{a}–\;\mathrm{RT}$$

(12) $$\Delta G_{d}^{{}^{\circ}}=-RT\;ln\;k_{d\;}$$

(13) $$\Delta S_{d}^{{}^{\circ}}=\frac{\Delta H_{d}^{{}^{\circ}}-\Delta G_{d}^{{}^{\circ}}}{T}\;\;$$

### 2.8. Characterization of Esterification Product

The purified EV was identified by gas chromatography (GC) and proton nuclear magnetic resonance (^1^H-NMR) spectroscopy. The gas chromatograph (Nexis GC-2030, Shimadzu, Kyoto, Japan) is equipped with a flame ionization detector (FID), and an ultra-inert capillary column 190915-433UI HP-5MS (30 m × 0.25 mm × 0.25 μm) was used to establish the molecular weight of the produced EV. The initial injector and detector temperatures were 250 °C and 260 °C, respectively, while the helium carrier gas was held at 1.0 mL/min under constant pressure. A 0.5 μL filtered sample was introduced (split ratio of 1/20) and eluted at 60 °C (held for 2 min) before increasing the temperature to 260 °C (3 °C/min). The analysis was triplicated, and each component is expressed as the percentage of the total peak area. Next, the ^1^H-NMR spectra of EV were taken on the NMR spectrometer (Avance II 400 MHz/54 mm, Bruker BioSpin, Fällanden, Switzerland) equipped with a 5 mm inverse probe. A 300 μL aliquot of the sample was dissolved in deuterated chloroform (CDCl_3_), injected, and analyzed at 278 K. The spectrum was collected at 128 scans in 2 h.

### 2.9. Statistical Analysis

The IBM SPSS software (Version 20.0, Chicago, IL, USA) was employed to analyze all triplicated experimental data. The Shapiro-Wilk tests showed normally distributed data, thereby requiring analysis of variance (ANOVA) repeated measurement to determine the statistical differences among mean values. Here, a *p*-value < 0.05 was taken as significant, and each datum is expressed as mean ± standard deviation.

## 3. Results and Discussion

### 3.1. Characterization of Supports and Biocatalyst

#### 3.1.1. Chemical Composition and Microcrystalline Structure Analysis

**Figure 1:**
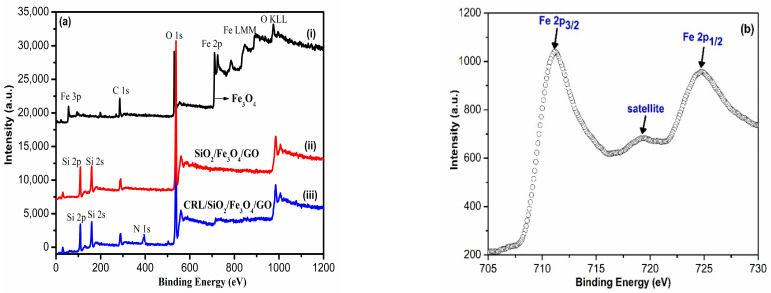

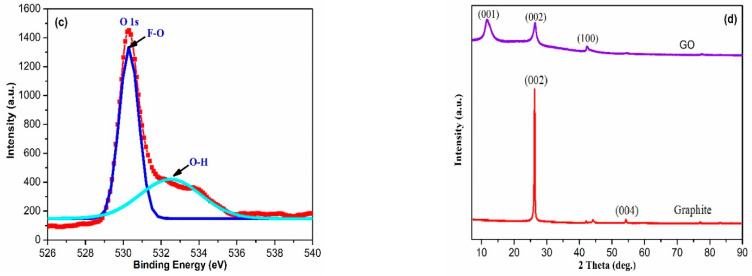
(**a**) XPS broad scan spectra of Fe_3_O_4_, SiO_2_/Fe_3_O_4_/GO, and CRL/SiO_2_/Fe_3_O_4_/GO, (**b**,**c**) the high-resolution spectrum of Fe 2p and O 1s regions of Fe_3_O_4_, respectively, and (**d**) XRD patterns of GO and pristine graphite.

XPS spectra between 0 and 1200 eV were collected and used to determine the samples’ elemental composition and oxidation states. Figure 1a represents the broad scan XPS spectra of Fe_3_O_4_, SiO_2_/Fe_3_O_4_/GO, and CRL/SiO_2_/Fe_3_O_4_/GO. The XPS spectrum of the Fe_3_O_4_ nanoparticles showed the existence of Fe and O in the sample (Figure 1a(i)). As seen, the binding energy peaks at 285, 532, and 711 eV were attributed to C 1s, O 1s, and Fe 2p, respectively (Figure 1a(i)) [30]. The presence of Fe and O elements in the sample suggested that the co-precipitation reaction successfully prepared the Fe_3_O_4_ nanoparticles.

In Figure 1a(ii), the peaks at 109 eV and 160 eV corresponded to the Si 2p and Si 2s of SiO_2_. The peak for Fe was not detected in the SiO_2_/Fe_3_O_4_/GO nanocomposite spectra, indicating the successful coating of the Fe_3_O_4_ nanoparticles with SiO_2_. In addition to the Si 2p, Si 2s, C 1s, and O 1s peaks at 108, 159, 290, and 534 eV, the spectrum of CRL/SiO_2_/Fe_3_O_4_/GO (Figure 1a(iii)) further revealed the presence of nitrogen as the N 1s peak at 398 eV, which implies covalently bound CRL molecules to the support. In addition, the intensities of the O 1s peaks for the SiO_2_/Fe_3_O_4_/GO support and CRL/SiO_2_/Fe_3_O_4_/GO catalyst were relatively higher compared to the Fe_3_O_4_ nanoparticles. This can be explained by the incorporation of more oxygen-rich oxides (SiO_2_, GO) to the Fe_3_O_4_ or the conjugation of APTES, GL, or CRL to the support by covalent bonding. Figure 1b presents the high-resolution XPS spectrum of Fe in the 2p region. The binding energy for pure Fe_3_O_4_ nanoparticles detected at 711.2 eV and 724.8 eV corresponded to the Fe 2p_3/2_ and Fe 2p_1/2_ of Fe_3_O_4_. A peak at 719.3 eV was assigned to the satellite peak for 2p_3/2_ [30] for trace amounts of Fe_2_O_3_ nanoparticles. The deconvoluted form in the O 1s spectrum for peaks at 530.28 and 532.45 eV were ascribed to the Fe-O bonds of Fe_3_O_4_ (Figure 1c) and residual oxygen-containing groups (i.e., O-H). The outcome seen here agrees with reported values for Fe_3_O_4_ in MGO/CRL biocatalyst [31].

The XRD diffractogram that proves the conversion of pristine graphite to GO is seen in the characteristic 2θ at 11. 67° and 42.19° peaks in the GO sheet for C-H stretches (Figure 1d) [32] that contrasted with the sharp, prominent peak (002) at 2θ = 26.6° for graphite (Figure 1d). The diffractogram of GO revealed two broad peaks at 11.7°, 26.5°, and a minor peak at 42.3°, corresponding to the (001), (002), and (100) diffraction planes [33]. The oxygenated groups on GO appeared as new diffraction peaks, which affirmed its successful synthesis from graphite [1].

#### 3.1.2. Textural Properties and Pore Structure Analysis

**Figure 2:**
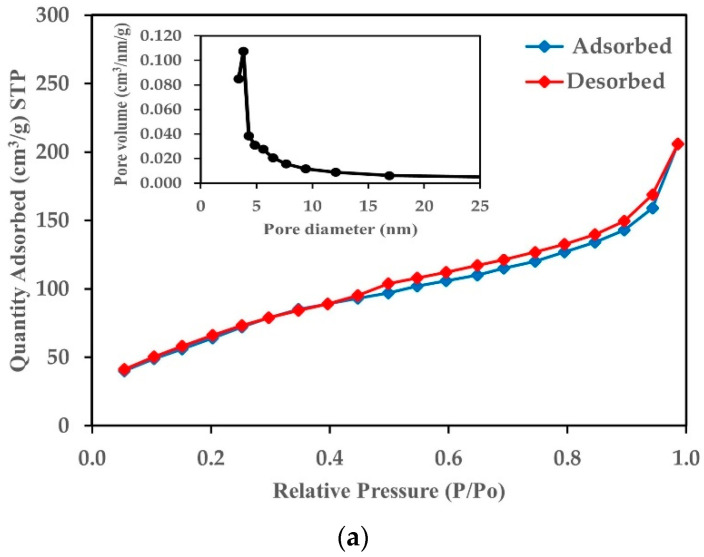

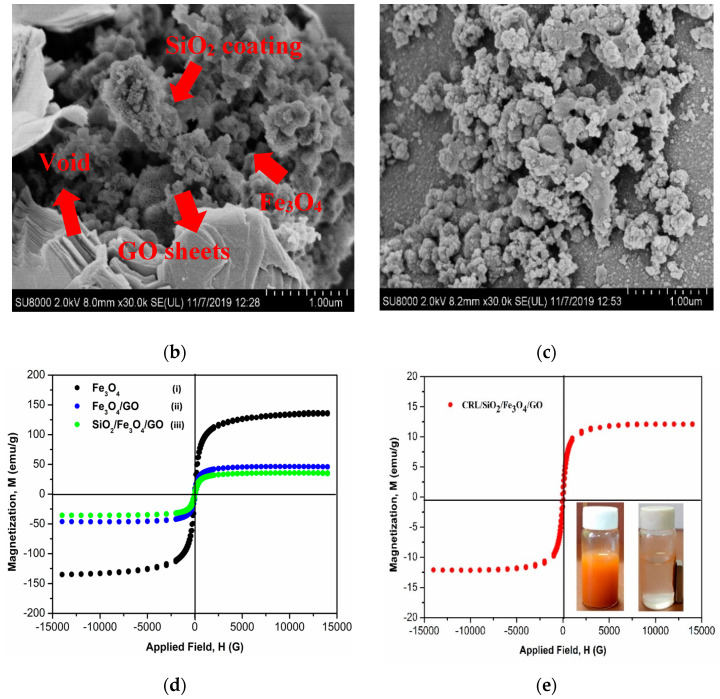
(**a**) N_2_ adsorption–desorption isothermal curve and insert pore distribution of GL-A-SiO_2_/Fe_3_O_4_/GO support; (**b**,**c**) FESEM micrographs of SiO_2_/Fe_3_O_4_/GO and CRL/SiO_2_/Fe_3_O_4_/GO (30,000× magnification); (**c**,**d**) Magnetization curves depicting the magnetic properties for (i) Fe_3_O_4_, (ii) Fe_3_O_4_/GO, (iii) SiO_2_/Fe_3_O_4_/GO, and (**e**) CRL/SiO_2_/Fe_3_O_4_/GO biocatalyst.

Figure 2a illustrates the N_2_ adsorption/desorption plot for the GL-A-SiO_2_/Fe_3_O_4_/GO support. The support exhibited an IUPAC type IV isotherm shape with a type H3 hysteresis loop at *P/P_o_* 4.3–1.0, suggesting well-formed mesopores [34]. The pore distribution (insert in Figure 2a), principally concentrated between 3.86 and 10 nm with a peak centered at 4 nm, supported the mesoporosity of GL-A-SiO_2_/Fe_3_O_4_/GO modified support. The 0.175 cm^3^/g BJH pore volume, 2.68 nm average pore diameter, and 260.87 m^2^/g BET specific surface area indicate the surface of GL-A-SiO_2_/Fe_3_O_4_/GO as a suitably large platform to form covalent bonds with CRL [1].

#### 3.1.3. Surface Morphological and Microstructural Analysis

Figure 2b,c illustrates the FESEM micrographs of the as-prepared ternary SiO_2_/Fe_3_O_4_/GO nanocomposite and the CRL/SiO_2_/Fe_3_O_4_/GO biocatalyst. As shown in Figure 2b, due to the large surface area of SiO_2_/Fe_3_O_4_/GO, characteristic wrinkled, layered structures with large void spaces were visible on the GO surface. The Fe_3_O_4_ nanoparticles appeared well-distributed and embedded within the graphene sheets and coated by layers of SiO_2_ [35]. Thus, the magnetic graphene-based silica nanocomposite support for CRL immobilization was successfully fabricated. As depicted in Figure 2c, the immobilization of CRL on the surface of the GL-A-SiO_2_/Fe_3_O_4_/GO support produced the CRL/SiO_2_/Fe_3_O_4_/GO biocatalyst. The surface was densely covered with irregularly shaped whitish globules likely to be protein aggregates of CRL. This observation correlates well with the results of *Rhizomucor miehei* lipase immobilized on chitosan-graphene oxide beads [23].

#### 3.1.4. Magnetic Behavior Analysis

Next, VSM characterization showed S-like shaped magnetization hysteresis curves for magnetic properties of (i) Fe_3_O_4_, (ii) Fe_3_O_4_/GO, and (iii) SiO_2_/Fe_3_O_4_/GO samples (Figure 2d), and CRL/SiO_2_/Fe_3_O_4_/GO (Figure 2e), corresponding to saturation magnetizations (Ms) 137.64 emu/g, 46.46 emu/g, 35.67 emu/g, and 12.12 emu/g. The lack of hysteresis in all magnetization curves (Figure 2d(i–iii)) with near-zero magnetic coercivity and retentivity validated the absence of residual magnetization when the external magnetic field was removed [36]. The superparamagnetic role of Fe_3_O_4_ in SiO_2_/Fe_3_O_4_/GO was evident in the magnetic separation of the catalyst (Figure 2e). The Fe_3_O_4_ revealed a remarkable Ms of ≈138 emu/g (>≈92 emu/g, Ms <≈140 emu/g) [37,38], but the Ms was predictably lower for Fe_3_O_4_/GO (46.46 emu/g). The declined superparamagnetism seen here was due to the presence of reduced GO nanosheets in Fe_3_O_4_/GO. Likewise, the lower Ms value for CRL/SiO_2_/Fe_3_O_4_/GO (12.12 emu/g) was due to several surface modifications on the Fe_3_O_4_ nanoparticles with non-magnetic materials during the support and CRL/SiO_2_/Fe_3_O_4_/GO preparations [36], plus when the biocatalyst was heated and washed in an aqueous solution [37]. However, the Ms of the CRL/SiO_2_/Fe_3_O_4_/GO biocatalyst was adequate for magnetic recovery from the reaction system (Figure 2e). The superparamagnetic property of SiO_2_/Fe_3_O_4_/GO is critical for rapid magnetic separation of the immobilized CRL from the reaction media (insert digital photographs) (Figure 2e). As can be seen, complete catalyst recovery was achieved within 45 s by placing an external magnet on the side of the glass bottle (Figure 2e).

#### 3.1.5. Chemical Composition and Functional Group Analysis

**Figure 3:**
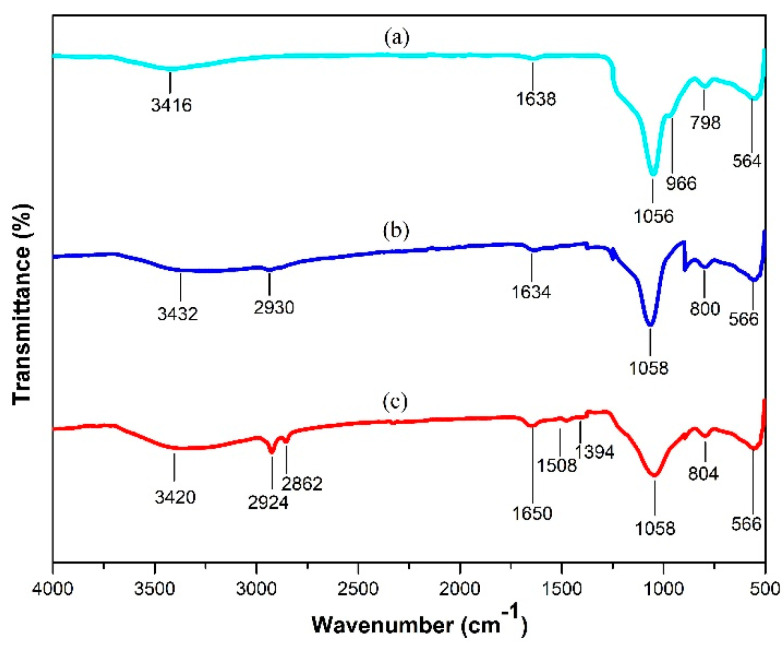
FTIR spectra of (**a**) SiO_2_/Fe_3_O_4_/GO, (**b**) GL-A-SiO_2_/Fe_3_O_4_/GO, and (**c**) CRL/SiO_2_/Fe_3_O_4_/GO.

The FTIR spectra of SiO_2_/Fe_3_O_4_/GO, GL-A-SiO_2_/Fe_3_O_4_/GO, and CRL/SiO_2_/Fe_3_O_4_/GO were acquired in ATR mode to identify the existence of distinct functional groups in the composites and biocatalyst (Figure 3). As shown in Figure 3a, the peaks for the main functional groups of Fe_3_O_4_, SiO_2_, and GO were clearly visible in the SiO_2_/Fe_3_O_4_/GO ternary nanocomposite. The absorption peak at 564 cm^−1^ agreed with the stretching mode of Fe–O bonds in Fe_3_O_4_. Conversely, the peaks centered at 966 cm^−1^, 798 cm^−1^ (Si-O-Si), and 1056 cm^−1^ (Si-O-Si) were ascribed to bending vibration, symmetric, and asymmetric stretching vibrations of silanol and siloxane groups of SiO_2_ particles [1]. The carbonyl group (C=O) of GO appeared at 1638 cm^−1^, whereas a broad peak located at 3416 cm^−1^ depicted the H-O-H bending vibration mode of loosely bonded water molecules. Similar peaks were also observed by Xie et al. [2] for CRL immobilized onto a magnetic graphene oxide nanocomposite.

New peaks were detected in the spectrum of GL-A-SiO_2_/Fe_3_O_4_/GO support (Figure 3b). A peak due to the C-H stretching mode of the methylene group in APTES and GL can be seen at 2930 cm^−1^, whereas that at 1634 cm^−1^ was attributed to the stretching vibrations of C=N bonds. The absorption peaks for C-H and C=N bonds in GL-A-SiO_2_/Fe_3_O_4_/GO (Figure 1b) pointed to covalent bound APTES at the surface of SiO_2_/Fe_3_O_4_/GO nanocomposite. The related bonds created the necessary activated surface groups and GL moieties for CRL covalent binding to SiO_2_/Fe_3_O_4_/GO. Figure 3c shows the CRL/SiO_2_/Fe_3_O_4_/GO spectrum, where multiple peaks at 1650 cm^−1^, 1508 cm^−1^, and 1394 cm^−1^ matched the stretching vibrations of C=N and C=O (Amide I) bonds, the bending mode of N-H (Amide II) bonds, and the stretching vibrations of C-C, C-N, or the bending vibration of N-H (Amide III) bonds, respectively [6]. Generally, amides I, II, and III peaks are valuable indicators to confirm the presence of an enzyme and its successful immobilization on support. Accordingly, the overall FTIR spectral data demonstrate a successful fabrication of the SiO_2_/Fe_3_O_4_/GO nanocomposite and the covalent immobilization of CRL, thus corroborating the XPS and VSM results.

### 3.2. Effect of Reaction Time on EV Synthesis

**Figure 4:**
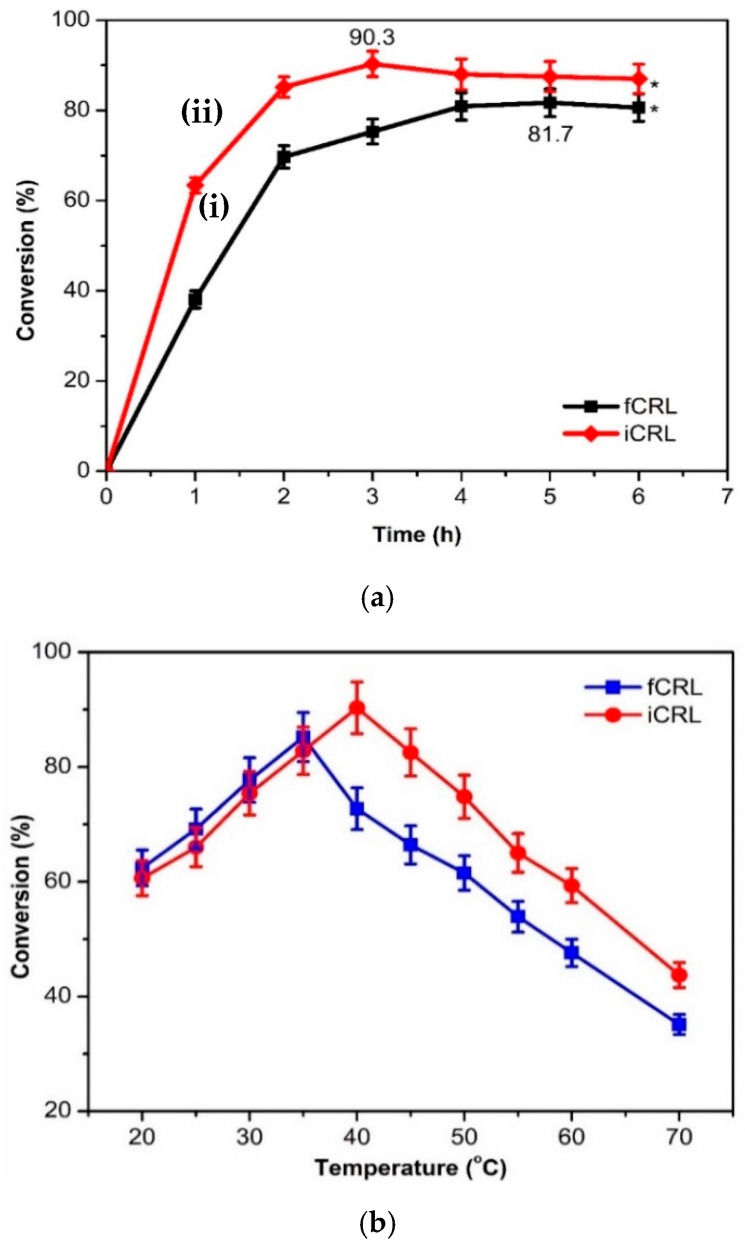
(**a**) The effect of reaction time on the synthesis of EV catalyzed by free CRL and CRL/SiO_2_/Fe_3_O_4_/GO biocatalysts (3 mg/mL, 40 °C, acid/butanol molar ratio 1:2, 200 rpm). * Independent *t*-test revealed a significant difference between the effects of reaction time to affect the percentage conversion of EV (*p* < 0.05), and (**b**) the thermal stability of free CRL and CRL/SiO_2_/Fe_3_O_4_/GO biocatalysts in the esterification synthesis of EV 3 mg/mL, acid/ethanol molar ratio 1:2, 200 rpm, 3 h.

Reaction time is an excellent measure of catalytic performance, in which durations vary for different enzymes to achieve maximum yields of different esters. Figure 4a presents the optimal duration for both free CRL and CRL/SiO_2_/Fe_3_O_4_/GO to catalyze high yields of EV. Both lipases achieved the highest percentage of EV before their activity began to plateau after 3 h. Free CRL catalyzed increased conversion of EV with increasing reaction time from 1 to 5 h (81.7%, *p* < 0.05) (Figure 4a(i)). The CRL/SiO_2_/Fe_3_O_4_/GO demonstrated a similar trend but reached the highest EV earlier at 3 h (90.3%, *p <* 0.05) (Figure 4b(ii)). The outcome was seen to support the significantly improved CRL/SiO_2_/Fe_3_O_4_/GO performance over the free CRL (*p* < 0.05). Thus, this outcome verified the biogenic SiO_2_/Fe_3_O_4_/GO nanocomposite’s feasibility to improve the catalytic activity of the immobilized CRL.

Pertinently, the notable improvement in catalytic activity could be linked to multipoint interactions between the CRL molecules with the GL-A-SiO_2_/Fe_3_O_4_/GO support. These addition interactions are known to rigidify the immobilized lipase structure to resist premature denaturation better [39]. Conversely, the plateau observed for CRL/SiO_2_/Fe_3_O_4_/GO after 3 h was presumably due to biocatalyst damage resulting from mechanical stress due to prolonged magnetic stirring [1]. In the case of free CRL, the percentage of produced EV declined beyond the 5 h duration. This might be due to surplus produced water, which is the by-product of esterification. Hence, the subsequent studies used 3 h as the optimal esterification time for CRL/SiO_2_/Fe_3_O_4_/GO.

### 3.3. Operational Stability Studies

#### 3.3.1. Thermal Stability and Half-Life

The effect of temperature on EV synthesis by free CRL and CRL/SiO_2_/Fe_3_O_4_/GO was investigated between 20 and 70 °C, and the results are depicted in Figure 4b. Both forms of biocatalysts displayed a similar trend of EV production from 20 to 40 °C. The percentage conversions of the ester for free CRL and CRL/SiO_2_/Fe_3_O_4_/GO increased with increasing temperatures up to 35 °C and 40 °C, respectively. However, a higher EV conversion was noted for the CRL/SiO_2_/Fe_3_O_4_/GO, occurring at a higher temperature of 45 °C (90.3%). This outcome also implied the higher thermal stability of CRL/SiO_2_/Fe_3_O_4_/GO than free CRL (85.2% at 40 °C). The superior thermal stability of the former over the free CRL could be ascribed to additional stabilization on the immobilized CRL structure by intermolecular covalent bonds to the SiO_2_/Fe_3_O_4_/GO support [8]. Plus, the SiO_2_/Fe_3_O_4_/GO shields the immobilized CRL against the denaturing effects of high temperatures [40]. The CRL/SiO_2_/Fe_3_O_4_/GO was more robust and retained 44% activity compared with only 35% in the free CRL, both at 70 °C.

**Figure 5:**
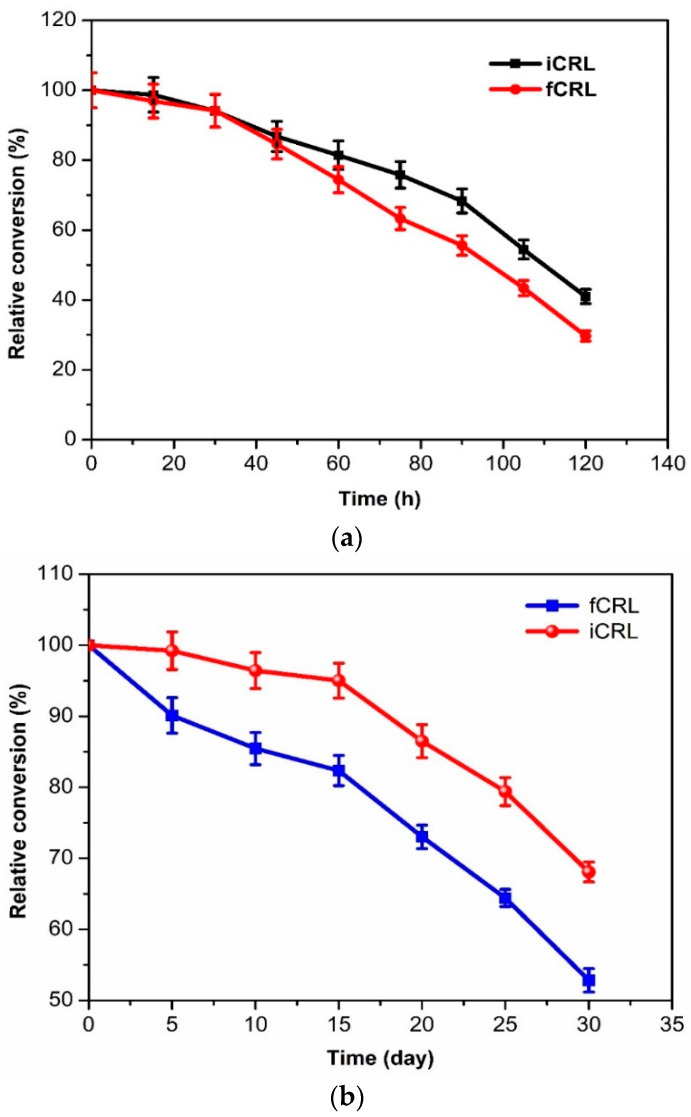
(**a**) Half-life of free CRL and CRL/SiO_2_/Fe_3_O_4_/GO biocatalysts at 50 °C, and (**b**) storage stability of free CRL and CRL/SiO_2_/Fe_3_O_4_/GO biocatalysts at room temperature in the esterification synthesis of EV (3 mg/mL, 40 °C acid/ethanol molar ratio 1:2, 200 rpm, 3 h).

Figure 5a denotes data for the half-life assessment on free CRL and CRL/SiO_2_/Fe_3_O_4_/GO, done at 50 °C and monitored for 130 h and 145 h, respectively. Progressively increasing the reaction duration from 15 to 120 h saw a decline in EV productions in reactions catalyzed by free and immobilized CRL (Figure 5a). The CRL/SiO_2_/Fe_3_O_4_/GO retained 45% of its original activity, which was a notable 15% higher than free CRL (30%). The data seen here corroborated the shielding effect of the SiO_2_/Fe_3_O_4_/GO support on the immobilized CRL. It has been documented that the protein structures of immobilized CRL molecules are safeguarded against premature unfolding, although only in some areas of the enzyme molecule [12]. Likewise, the additional support/protection from the SiO_2_/Fe_3_O_4_/GO to a certain degree disrupts the native CRL protein unfolding pathway, resulting in enhanced lipase stability. Conversely, the second fraction without the support’s protection behaves as a soluble enzyme; hence, it is quickly deactivated by higher temperatures [12].

#### 3.3.2. Short-Term Storage Stability

The high costs of replacing enzymes and the time it takes to immobilize them have created demands for enzymes with extended durations of storage stability. Figure 5b illustrates the data of storage stability measurement for free CRL and CRL/SiO_2_/Fe_3_O_4_/GO, which is stored at room temperature for four weeks. The CRL/SiO_2_/Fe_3_O_4_/GO showed higher storage stability than the free CRL, with the former retaining ≈68% activity compared to 53% in the free CRL. The findings again supported the SiO_2_/Fe_3_O_4_/GO nano support’s efficacy in enhancing the lipase’s robustness. As can be seen, the immobilized CRLs could better retain their catalytic activity and stability due to additional stabilization of their protein structure through multiple interactions with the SiO_2_/Fe_3_O_4_/GO support. The outcome seen here also corroborates the findings of earlier researchers [41,42].

#### 3.3.3. Regeneration Study

**Figure 6:**
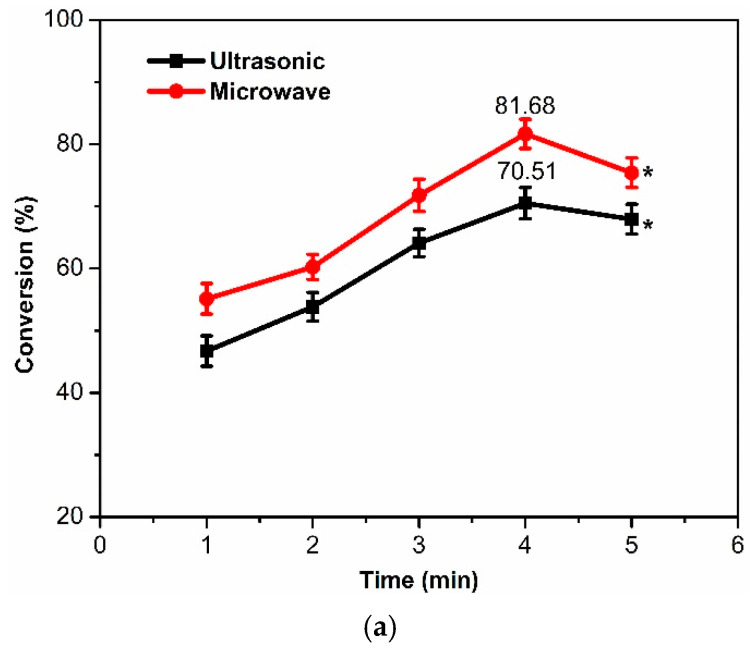

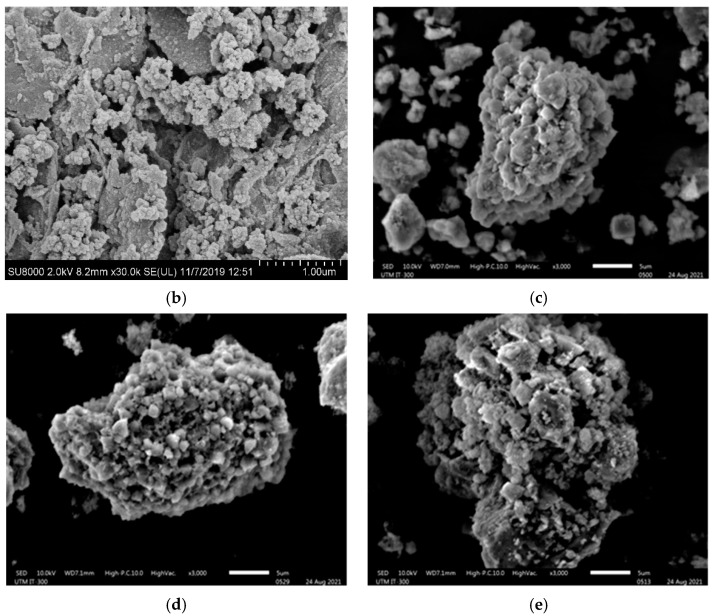
(**a**) Esterification activity of regenerated CRL/SiO_2_/Fe_3_O_4/_GO after ultrasonication and microwave treatments. Next, * Independent *t*-test revealed a significant difference between the effects of reaction time to affect the percentage conversion of EV (*p* < 0.05), the SEM micrographs of (**b**) fresh, (**c**) reused, (**d**) ultrasonicated, and (**e**) microwave-treated CRL/SiO_2_/Fe_3_O_4/_GO (3000× magnification).

Regeneration is an important step in extending a biocatalyst’s usefulness and could contribute to cost savings. Bearing this in mind, this study explored two kinds of regeneration steps on spent CRL/SiO_2_/Fe_3_O_4/_GO, which was previously reused for 11 consecutive esterification cycles. The spent biocatalyst was subjected to two different regeneration steps, ultrasonication and microwave, for treatment durations between 1 and 5 min. Then, the biocatalyst was suspended into fresh media and assayed for the esterification synthesis of EV under optimal conditions. In general, both biocatalyst regeneration treatments demonstrated a similar trend in EV synthesis for durations of 1 to 4 min and reached the maxima at 4 min before declining (Figure 6a).

As shown in Figure 6a, a maximum EV conversion of 70.51% was achieved for the ultrasonicated CRL/SiO_2_/Fe_3_O_4/_GO compared to 81.68% for the microwave-treated biocatalyst (*p* < 0.05). The observation proved that the latter was the better treatment to rejuvenate spent CRL/SiO_2_/Fe_3_O_4/_GO. From another viewpoint, the outcome also implied that both treatments were applicable in rejuvenating the spent catalyst. Nonetheless, beyond 4 min, the microwave-treated CRL/SiO_2_/Fe_3_O_4_/GO activity dropped sharply to 75.41%. This decline was presumably due to lipase deactivation from prolonged exposure to microwave heating, as previously documented by another study that attempted to rejuvenate the ZnO-immobilized horseradish peroxidase by microwave irradiation [43]. They also reported that the treatment was better in dislodging the build-up of deposits or contaminants blocking the catalytic sites without compromising the enzyme’s active conformation.

Figure 6b shows the SEM micrograph of the freshly prepared CRL/SiO_2_/F_3_O_4_/GO. As can be seen, the immobilization of CRL on the GL-A-SiO_2_/Fe_3_O_4_/GO surface resulted in a denser-looking exterior of the CRL/SiO_2_/Fe_3_O_4_/GO biocatalyst, dotted with irregularly shaped whitish globules, which was believed to be CRL. Figure 6c–e depict SEM micrographs of, reused, alongside ultrasonic and microwave regenerated CRL/SiO_2_/F_3_O_4_/GO. The external morphology of the reused biocatalyst after 11 cycles showed numerous deposits and fragmented surfaces, which are evidence of blockages to CRL active sites. Mechanical-related damages, starkly contrasted with the morphology of the freshly prepared CRL/SiO_2_/F_3_O_4_/GO, were also observed. The deposits, possibly from trapped substrates or the enzymatically synthesized EV, obscured the entry of new substrates to the active sites of CRL/SiO_2_/F_3_O_4_/GO. This justifies the reduced percentage of EV (≈48%) with further continued use of the biocatalyst.

After the ultrasonication (Figure 6d) and microwave (Figure 6e) treatments, the deposits over the surface of CRL/SiO_2_/F_3_O_4_/GO were visibly reduced, suggesting fewer obstructions to the lipase active sites. Both treatments on CRL/SiO_2_/F_3_O_4_/GO were highly effective, as corroborated by the high percentage of synthesized EV, which rose from less than 50% to reach a satisfactory maximum at 70.51% and 81.68%, respectively. A similar observation was reported by Jaiswal et al. [44] for *Candida antartica* B lipase immobilized on polyacrylate beads.

### 3.4. Thermodynamic Study

#### 3.4.1. Kinetic Rate Constant and Activation Energy

**Figure 7:**
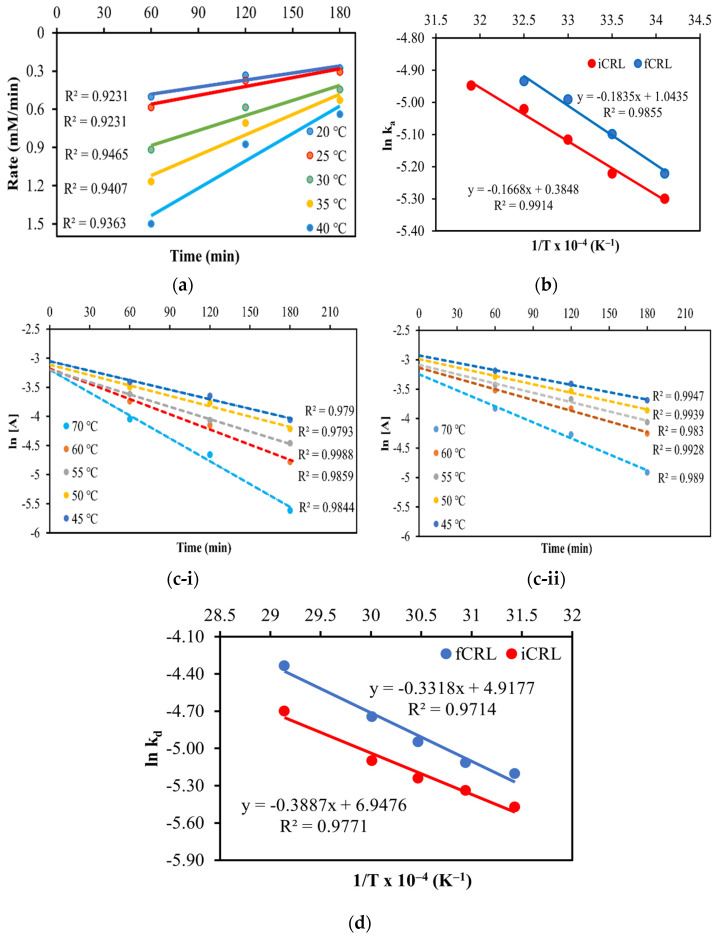
(**a**) First-order plot for the thermal activation rate constants ($k_{a}$) at various temperatures, (**b**) Arrhenius plot for the activation energy for free CRL (fCRL, blue), and CRL/SiO_2_/Fe_3_O_4_/GO (iCRL, red), (**c-i**,**c-ii**) First-order plots for thermal deactivation rate constants $\left(k_{d}\right)$, and (**d**) Arrhenius plot for the deactivation energy of free CRL (fCRL, blue), and CRL/SiO_2_/Fe_3_O_4_/GO (iCRL, red), respectively.

The rate constant ($k_{a})\;$ for the CRL/SiO_2_/Fe_3_O_4_/GO was determined by plotting the reaction rate against time at various temperatures (20 °C, 25 °C, 30 °C, 35 °C, 40 °C) (Figure 7a). In this study, the temperature-influenced enzymatic esterification followed the first-order kinetic, producing correlation coefficients between 0.9231 and 0.9465. Activation energy ($E_{a}$) is generally a crucial parameter affecting enzyme activity. That said, the $E_{a}$ values for both lipases were calculated from the regressions of Arrhenius plots (Figure 7b). As seen, $E_{a}$ values for free CRL and CRL/SiO_2_/Fe_3_O_4_/GO were 15.26 kJ/mol and 13.87 kJ/mol at their optimal temperatures of 35 °C and 40 °C, respectively (Table 1). Interestingly, the immobilization of CRL onto the SiO_2_/Fe_3_O_4_/GO support reduced the $E_{a}$, yielding larger EV conversions over the free CRL.

It was apparent that stabilizing the CRLs through covalent bonds with the SiO_2_/Fe_3_O_4_/GO directly reduced the energy barrier of the immobilized CRL by 1.39 kJ/mol. The added intermolecular bonds between the lipase and support justified the SiO_2_/Fe_3_O_4_/GO’s ability to convert more EV than the free CRL, as the latter was less susceptible to deactivation. The study findings were consistent with the following results published in the following literature. Bodakowska-Boczniewicz and Garncarek [45] observed a similar outcome on the $E_{a}$ of free Naringinase (25.18 kJ/mol) in comparison to its immobilized form on magnetic polysaccharide support (17.66 kJ/mol). In another study, Liu et al. [46] similarly reported higher $E_{a}$ for free *Burkholderia cepacia* lipase (25.18 kJ/mol) than the immobilized form (17.66 kJ/mol).

#### 3.4.2. Deactivation Rate Constant and Deactivation Energy

The thermal deactivation constants ($k_{d}$) and the deactivation energy ($E_{d}$) were calculated for the free CRL and CRL/SiO_2_/Fe_3_O_4_/GO from 45 to 70 °C (Figure 7c-i,ii,d). As shown, the correlation coefficients (R^2^ > 0.97) obtained from the regressions of their Arrhenius plots revealed that the thermal deactivation constants for both free CRL and CRL/SiO_2_/Fe_3_O_4_/GO obeyed the first-order kinetic laws. Plotting ln $k_{d}\;$ versus temperature using non-linear regressions showed the estimated deactivation energy ($E_{d}$), as described for $E_{d}$ (Figure 7d). $E_{d}$ values for the free CRL and CRL/SiO_2_/Fe_3_O_4_/GO were found to be 27.7 kJ/mol and 32.32 kJ/mol, respectively (Table 1). Pertinently, $E_{d}$ denotes the initial energy requirement for the onset of denaturation. The data suggest that the additional intermolecular covalent bonds offered between the SiO_2_/Fe_3_O_4_/GO support and the CRLs prevented the lipase protein from prematurely unwinding at higher temperatures. As a result of the higher $E_{d}$ value in CRL/SiO_2_/Fe_3_O_4_/GO than the free CRL, it was clear that the immobilized lipase was more stable and robust enough to withstand thermal denaturation than its free counterpart [47]. Therefore, the findings were comparable to an earlier study on CRL immobilized on SiO_2_-MNPs support [8].

#### 3.4.3. Thermodynamics Parameters

According to Elias et al. [48], kinetic and thermodynamic information could provide valuable insights into enzymes’ reaction rates. The data are also useful for comparing the activity and heat stability of different enzymes. In this study, the $k_{d}$ value of free CRL is substantially higher (0.0055–0.0131 min^−1^) than CRL/SiO_2_/Fe_3_O_4_/GO (0.0042–0.0091 min^−1^), thus supporting the latter’s higher thermal stability (Table 2). The results also corroborated the longer half-life of CRL/SiO_2_/Fe_3_O_4_/GO (145 h) over the free CRL (130 h) (Section 3.3.1). The same trend of lower $k_{d}\;$ values of the immobilized CRL were observed from temperatures 45 to 70 °C, signifying the thermal protective effects of the SiO_2_/Fe_3_O_4_/GO support on the immobilized CRL.

The term half-life ($t_{1/2}$) defines the time that leads to a 50% reduction of catalyst activity. In this study, it was shown that the $t_{1/2}$ of CRL/SiO_2_/Fe_3_O_4_/GO increased between ≈1.25 and ≈1.82 fold compared to the free CRL for 45–70 °C (Table 2). The trend seen here conveyed the higher thermal resistance of CRL/SiO_2_/Fe_3_O_4_/GO over the free CRL due to additional intermolecular covalent bonds stabilization on the CRLs by the SiO_2_/Fe_3_O_4_/GO support. Correspondingly, the increase in D- and SF values further affirmed the SiO_2_/Fe_3_O_4_/GO nano support’s efficacy and suitability for improving the thermal stability of CRL (Table 2). The elevated thermal stability of CRL/SiO_2_/Fe_3_O_4_/GO seen here is a good indicator that the biocatalyst might have commercial significance in catalyzing synthetic reactions under industrial settings. Similar findings for *Rhizomucor miehei* lipase immobilized on magnetic nanoparticles were also documented [42].

Other thermodynamic parameters, such as the standard enthalpy of denaturation (Δ$H_{d}^{{}^{\circ}}$), standard entropy of denaturation (Δ$S_{d}^{{}^{\circ}}$), and Gibbs free energy (Δ$G_{d}^{{}^{\circ}}$) were also calculated in the temperature range of 45–70 °C, and Table 2 enlists the results. The Δ$H_{d}^{{}^{\circ}}\;$ values of CRL/SiO_2_/Fe_3_O_4_/GO marginally reduced from 24.96 to 24.75 kJ/mol^−1^ with increasing temperatures (45–70 °C), and the same goes for the free CRL, which declined from 29.67 to 29.46 kJ/mol^−1^. The consistently higher and positive enthalpic terms of CRL/SiO_2_/Fe_3_O_4_/GO over the free CRL again verified the lipase’s greater resistance to thermally-related denaturation [1] after immobilization. The data also meant that higher energy is required to denature the CRL/SiO_2_/Fe_3_O_4_/GO protein than free CRL.

The literature indicated that Δ$G_{d}^{{}^{\circ}}$ represents a more reliable parameter to estimate and compare the stability of enzymes [8]. That said, the study found that Δ$G_{d}^{{}^{\circ}}\;$ values (≈13.0–15.0 kJ mol^−1^) for CRL/SiO_2_/Fe_3_O_4/_GO were consistently higher than the free CRL (≈12.0–14.0 kJ mol^−1^) at all tested temperatures (Table 2). The positive and improved Δ$G_{d}^{{}^{\circ}}$ of CRL post-immobilization onto the SiO_2_/Fe_3_O_4_/GO support also validated a non-spontaneous lipase denaturation [8]. In other words, the CRL/SiO_2_/Fe_3_O_4_/GO does not undergo rapid deactivation at temperatures above its optimum, which is a trend seen here that agreed with the notably longer half-life (165.04–76.17 °C min), higher Δ$H_{d}^{{}^{\circ}}\;$ (29.67–29.46 kJ mol^−1^), and lower $K_{d}$ (0.0042–0.0091 min^−1^) values compared to free CRL for temperatures 45–70 °C. In addition, the higher and positive values of $\Delta S_{d}^{{}^{\circ}}$ of the CRL/SiO_2_/Fe_3_O_4_/GO (47.68–46.83 J mol^−1^K^−1^) than free CRL (34.54–36.14 J mol^−1^K^−1^) conveyed a higher disordered state or randomness of the former (Table 2). This meant that the thermal denaturation of free CRL occurs via protein unfolding instead of aggregation [13,48].

### 3.5. Product Identification and Structural Elucidation of Ethyl Valerate

#### Gas Chromatography and Proton Nuclear Magnetic Resonance Analysis

**Figure 8:**
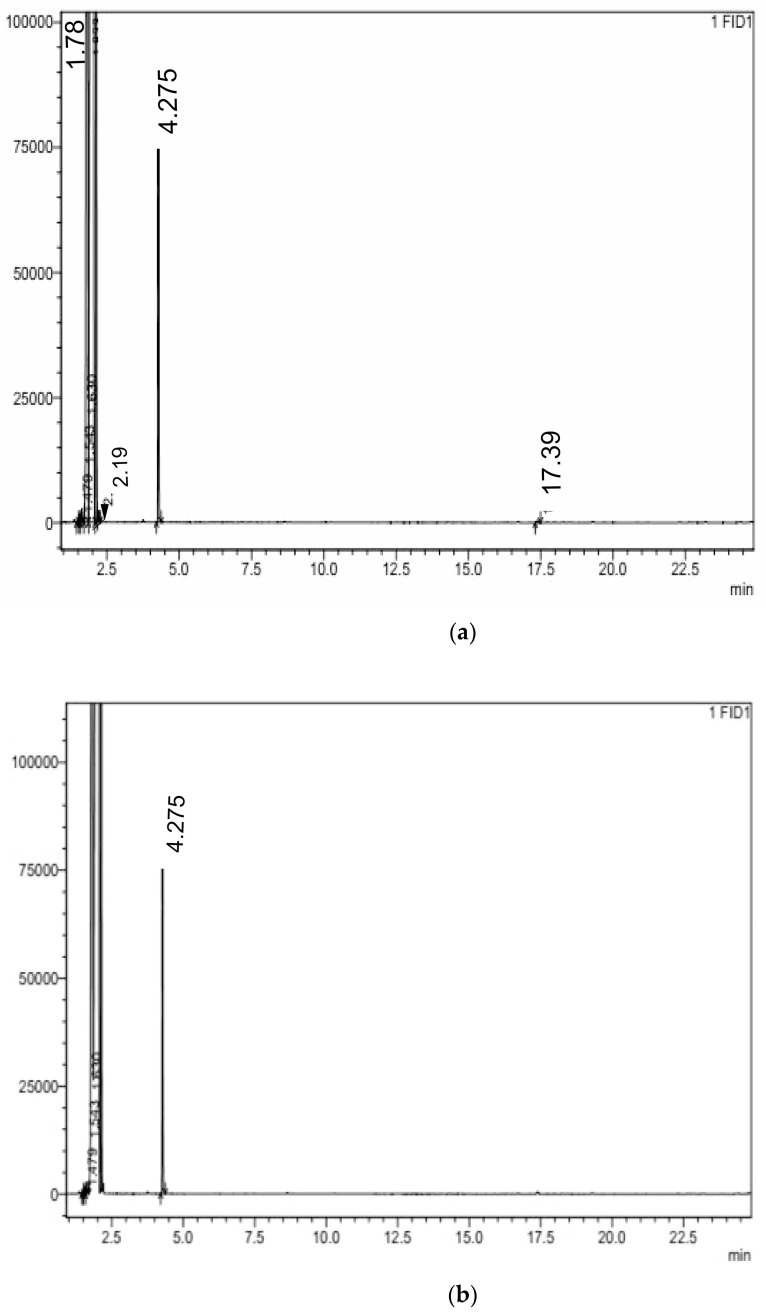
(**a**) GC of the enzymatically produced EV and (**b**) EV after purification.

The CRL/SiO_2_/Fe_3_O_4_/GO synthesized EV was validated by gas chromatography (GC) and proton nuclear magnetic resonance (^1^H-NMR) analysis. The boiling points of the withdrawn reaction components are as follows: ethanol (78.37 °C) < ethyl valerate (145.9 °C) < valeric acid (186.19 °C). As seen in Figure 8a, the solvent peak emerged at 1.78 min followed by ethanol (2.19 min), whereas EV and valeric acid were detected at 4.28 min and 17.39 min, respectively. After purification to obtain the pure EV, only a prominent peak of the pure ester was observed at 4.28 min (Figure 8b). This proved that EV was successfully synthesized by CRL/SiO_2_/Fe_3_O_4_/GO. The comparable retention time for the chromatogram of EV was also described in an earlier study [49].

**Figure 9:**
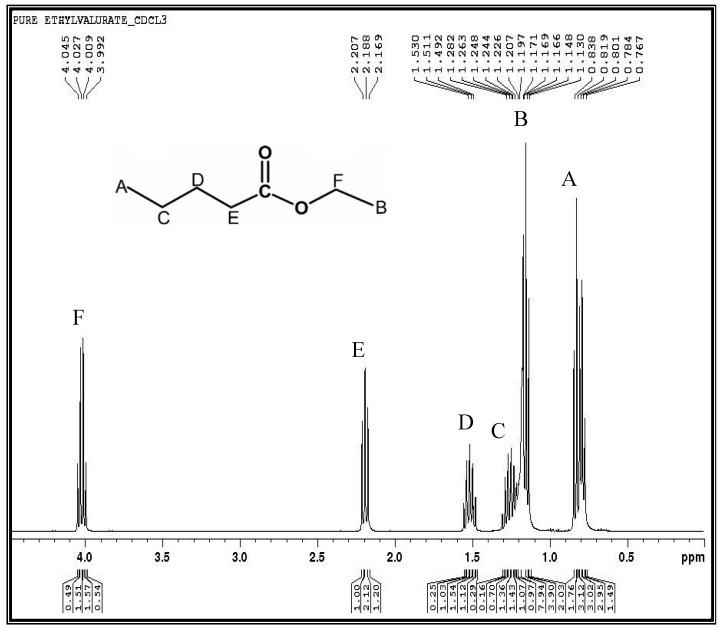
^1^H-NMR spectrum for the pure EV (valeric acid and ethanol molar ratio (1:2) at 3 h reaction).

The purified EV was further characterized by ^1^H-NMR spectroscopy to identify and confirm the product. In this assessment, the ^1^H-NMR spectrum of EV revealed six types of 14 hydrogen atoms, which were unique for EV (CAS: 539-82-2) (Figure 9). The signals with chemical shifts δ (ppm) at 0.84 (3H, triplet, —CH_3_) and 1.17 (3H, triplet, —CH_3_); 1.26 (2H, sextet, —CH_2_), 1.51 (2H, quintet, —CH_2_), and 2.19 (2H, triplet, —CH_2_); and 4.05 (2H, quartet, —OCH_2_) corresponded to methyl, methylene, and oxymethylene hydrogen atoms. Protons ‘A’ (δ = 0.84 ppm) and ‘B’ (δ = 1.17 ppm) appeared at lower magnetic fields. In comparison, proton ‘F’ of oxymethylene showed a chemical shift of δ = 4.05 ppm due to the de-shielding effect of the oxygen’s strong electronegativity. However, an intervening carbon reduced the de-shielding effect of oxygen on proton ‘E,’ to give a chemical shift at δ = 2.19 ppm compared to other methylene protons in EV. This is due to the rather high electronegativity of the adjacent C=O carbon. The electronegativity effect of heteroatom oxygen in EV decreased further down the chain. This effect was evident in the methylene protons’ C’ (δ = 1.26 ppm) and the weaker de-shielding effect, ‘D’ (δ = 1.51 ppm), thus explaining their lower chemical shifts. The results confirmed that EV was enzymatically produced in the reaction and the ^1^H-NMR data agreed with the GC-FID data.

## 4. Conclusions

The study demonstrates that the fabricated biogenic SiO_2_/Fe_3_O_4_/GO support effectively improved the catalytic and operational stability of immobilized CRL. The resultant biocatalyst successfully catalyzed higher yields of EV compared to free CRL. As revealed by the BET model, the GL-A-SiO_2_/Fe_3_O_4_/GO large specific surface area provided an adequate surface for multipoint conjugation with CRL. This added stabilization corroborated the greater stability and activity of the CRL/SiO_2_/Fe_3_O_4_/GO than its free form. The superior activity and stability of CRL/SiO_2_/Fe_3_O_4_/GO were echoed by the generally improved thermodynamic parameters of the biocatalyst. Values of *E_a_*, standard enthalpy, and the *E_d_* of CRL/SiO_2_/Fe_3_O_4_/GO were consistently lower than free CRL at all tested temperatures. In a nutshell, the findings pointed to the suitability of the combined biogenic inorganic-organic materials in the SiO_2_/Fe_3_O_4_/GO to improve the stability and activity of immobilized CRL. Thus, the CRL/SiO_2_/Fe_3_O_4_/GO is a potential industrial biocatalyst for producing other commercial esters.

## Figures and Tables

**Table 1:** Summary of activation and deactivation energies of free CRL and CRLSiO_2_/Fe_3_O_4_/GO.

Biocatalyst	$E_{a}\;(\mathrm{kJ}/\mathrm{mol})$	R^2^	$E_{d}\;(\mathrm{kJ}/\mathrm{mol})$	R^2^
Free *C. rugosa* lipase	15.26	0.9855	27.60	0.9714
CRL/SiO_2_/Fe_3_O_4_/GO	13.87	0.9914	32.32	0.9771

**Table 2:** Thermodynamic and thermal denaturation parameters for free CRL and CRLSiO_2_/Fe_3_O_4_/GO.

Parameter	Biocatalyst	Temperature				
		45 °C	50 °C	55 °C	60 °C	70 °C
$K_{d}$ (min^−1^)	Free CRL	0.0055	0.0060	0.0071	0.0087	0.0131
	CRLSiO_2_/Fe_3_O_4_/GO	0.0042	0.0048	0.0053	0.0061	0.0091
$t_{1/2}$ (min)	Free CRL	126.03	115.52	97.63	79.67	52.91
	CRLSiO_2_/Fe_3_O_4_/GO	165.04	144.41	130.78	113.63	76.17
SF	CRLSiO_2_/Fe_3_O_4_/GO	1.310	1.250	1.340	1.426	1.450
D-value (min)	Free CRL	418.65	383.76	324.31	264.66	175.77
	CRLSiO_2_/Fe_3_O_4_/GO	548.23	479.71	434.45	377.47	253.03
$\Delta H_{d}^{{}^{\circ}}\;$ (kJ mol^−1^)	Free CRL	24.96	24.92	24.87	24.83	24.75
	CRLSiO_2_/Fe_3_O_4_/GO	29.67	29.63	29.59	29.55	29.46
$\Delta G_{d}^{{}^{\circ}}$ (kJ mol^−1^)	Free CRL	13.79	13.76	13.51	13.25	12.35
	CRLSiO_2_/Fe_3_O_4_/GO	14.50	14.36	14.31	14.23	13.39
$\Delta S_{d}^{{}^{\circ}}$ (J mol^−1^K^−1^)	Free CRL	35.11	34.54	34.62	34.76	36.14
	CRLSiO_2_/Fe_3_O_4_/GO	47.68	47.25	46.56	45.99	46.83

## Data Availability

The raw/processed data required to reproduce these findings cannot be shared at this time as the data also form part of an ongoing study.
